# The association between infrapatellar fat pad volume and knee pain in non-obese postmenopausal women

**DOI:** 10.1016/j.ostima.2026.100465

**Published:** 2026-06-11

**Authors:** Nima Yazdankhah, Vahid Anwari, Siwen Liu, Emily Ha, Andy Kin On Wong

**Affiliations:** a Schroeder Arthritis Institute, University Health Network, Toronto, ON, Canada; b Joint Department of Medical Imaging, University Health Network, Toronto, ON, Canada; c Rehabilitation Sciences Institute, University of Toronto, Toronto, ON, Canada; d Dalla Lana School of Public Health, University of Toronto, Toronto, ON, Canada

**Keywords:** pQCT, MRI, Infrapatellar, Fat pad, Knee symptoms, Osteoarthritis, Tibiofemoral, Postmenopausal women, Non-obese

## Abstract

**Background:**

The infrapatellar fat pad (IPFP) is a significant source of pro-inflammatory cytokines and may be related to knee pain. The study aim was to determine the association between knee symptoms and each of IPFP size from knee MRI and density from peripheral (p) QCT knee images in non-obese postmenopausal women.

**Methods:**

Postmenopausal women 50–85 yrs old with a body mass index (BMI) < 30 kg/m^2^ and variable knee pain completed proton-density-weighted MRI and pQCT scans of the knee (one slice at the level of each compartment), where the IPFP was contoured to yield volumes (for MRI), areas and density (for pQCT). Knee symptoms were evaluated using the Intermittent and Constant Osteoarthritis Pain (ICOAP) and Knee Osteoarthritis Outcomes Score (KOOS) questionnaires, and OARSI physical function tests. Multivariable linear regression examined how IPFP metrics relate to these outcomes, accounting for age, BMI, use of pain medications, and Kellgren Lawrence (KL) score (≥2 vs <2).

**Results:**

Among 47 women (mean age: 61.2(8.82) yrs, BMI:22.5(3.2) kg/m^2^, KL score ≥2: 27.6%), a 1 cm^2^ smaller IPFP area from pQCT slices of the medial femoral compartment was associated with a 2.3% lower KOOS activities of daily living (ADL) score, a 4.7% higher ICOAP constant pain score, and a 3.9% higher ICOAP total pain score. A 1 mg/cm^3^ higher IPFP density from the pQCT slice of the lateral tibial compartment was associated with a 4.27% lower KOOS ADL score. Meanwhile, a 1 cm^3^ larger overall IPFP volume from MRI was associated with a 2% higher ICOAP intermittent pain score.

**Conclusions:**

A denser and thinner IPFP cross-sectional area but with larger overall volume - suggesting a long, dense, and skinny phenotype - may be a correlate of having more knee pain in postmenopausal women.

## Introduction

Knee osteoarthritis (OA) is a chronic, whole-joint disease marked by progressive deterioration of the knee joint which typically manifests as pain leading to disability, while other times remaining asymptomatic [1]. Known risk factors for knee OA and its associated pain include older age, female sex, and higher body mass index (BMI) [2,3]. Recent evidence is building on the relationship between periarticular fat distributions and knee symptoms in OA[4]. The infrapatellar fat pad (IPFP), also known as Hoffa’s fat pad, has long been recognized as an active intra-articular structure implicated in knee symptoms, including anterior knee pain, synovitis-related signal abnormalities, and OA-related structural change[5]. Rather than being a passive fat depot, the IPFP has mechanical, vascular, neural, and inflammatory features that may relate to symptoms through multiple pathways[6,7].

The IPFP is situated inferior and deep to the patella, and anterior to the femoral and tibial condyles [8]. A histological view of the fat pad shows lobules of adipocytes within white adipose tissue [9], but also fibroblasts producing extracellular matrix and signaling immune cells that trigger progression of OA [10]. The IPFP provides mechanical support for the patella and knee joint by reducing impact, friction, and pinching of the bones [9], as suggested by *Bohnsack* et al. who showed the IPFP’s function in stabilizing the knee at the extremes of flexion and extension (<20° and >100°) [11]. However, mechanical support is not its sole function. More research is proposing the additional influence of IPFP as a hub of innervation and vascularity that promotes inflammation and knee pain [9,10,12].

Adipocytes within IPFP secrete pro-inflammatory cytokines and growth factors to the knee joint, creating an environment that can fuel pain cascade mechanisms [10,13]. The IPFP is a richly innervated structure containing both sensory fibers, as demonstrated by a positive test showing homogeneous distribution of S-100, Substance P[9], and sympathetic fibers; revealed by positive Tyrosine Hydroxylase (TH) markers [3,14]. One study by *Lisowska* et al. has shown higher levels of Substance P in the IPFP to be correlated with chronic pain and inflammation in OA patients [15]. Given the homogeneity of Substance P distribution within the IPFP, it may be plausible to suggest that a larger volume of IPFP may correlate with higher Substance P levels.

Previous longitudinal studies have found that a larger cross-sectional area of the IPFP to be associated with reduced loss of the surrounding cartilage volume [2,14] However, few have examined IPFP volume’s associations with pain. Trauma to the fat pad manifests clinically as anterior knee pain, particularly after multiple microtrauma [9,16]. Increased volume and severe inflammation within the IPFP have been associated with severe pain. However, this evidence was uncovered in a population of overweight and obese men and women, where systemic fat could potentially influence this relationship [17].

Magnetic Resonance Imaging (MRI) visualizes soft tissue contrast by differentiating proton relaxation properties. Given fat's high proton content contrasting against that of surrounding bone, ligaments, and joint space, this modality can visualize IPFP morphology in 3-dimensions. Although CT is better calibrated to capture and quantify subchondral bone, it can also depict the presence of larger pools of fat such as the IPFP against a backdrop of surrounding higher density fibrocartilaginous tissues and muscle. By examining the density of IPFP, it may also be possible to evaluate the degree of fibrosis, an inflammation-linked [18] feature known to track with knee OA outcomes [23,26]. Single slice images acquired from pQCT, while impractical for volumetric data, yields size and density information at specific locations relative to the joint which may contribute differently to knee symptoms. The objective of this study was to determine how larger IPFP area/volume as measured on MRI versus peripheral (p) QCT, and IPFP density measured on pQCT, each relate to knee symptoms in non-obese postmenopausal women with evidence of knee OA. Knowing which region yields the strongest clinical correlation could inform future protocols and save time from performing extraneous scans. pQCT is also lower cost and requires shorter time to acquire compared to MRI, and given large MRI workloads in healthcare overburdening the system, pQCT may offer a reasonable alternative. Multi-modal imaging could further provide complementary information on a complex tissue like IPFP, allowing a more comprehensive exploration of its clinical value in the present study.

## Methods

**Study design:** This was a cross-sectional study with convenience sampling. Advertising for the study was posted on billboards around the University Health Network hospitals and in the community. **Recruitment:** Non-obese (self reported BMI <30 kg/m^2^), post-menopausal women between 50–85 years old were prospectively recruited. Those with rheumatoid arthritis, previous joint replacements, any renal diseases (including transplants), or contraindications to MR imaging (pacemaker, insulin pump, neurostimulator) were excluded. Participants were required to have self-reported mild to severe knee pain, or to have a diagnosis of knee OA. The study was conducted at the Toronto General Hospital Research Institute, and approved by the Research Ethics Board at the University Health Network (#17–5031). Eligible participants were enrolled into this Postmenopausal Knee Imaging of Intramedullary Pressure (PoKIMP) study.

**Knee symptoms (outcome):** The Knee Osteoarthritis and Outcome Score (KOOS) [19] and the Intermittent and Constant Osteoarthritis Pain (ICOAP) questionnaires [20] were used as valid semi-quantitative assessments of knee pain. The KOOS questionnaire surveyed Activities of Daily Life (KOOS ADL), Quality of Life (KOOS QoL), Sports & Recreational Activities (KOOS Sport/Rec), and Pain. Values range from 0 to 100 with lower values indicating worse outcomes. The ICOAP questionnaire consists of a 5-question constant pain component and a 6-question intermittent pain component – each item being rated from 0 to 4 with 4 representing extreme or frequent pain. Values from ICOAP were normalized to a similar 0 to 100 scale with higher values indicating worse outcomes [19,20]. Three tests were used to assess physical function as recommended by the Osteoarthritis Research Society International (OARSI): a 30 s chair stand test (repetitions), a 40 m walk test (time, s), and a 9-step stair climb test (time, s).

**Image acquisition:** Images of the single knee exhibiting greater pain were acquired using a 3.0T Siemens Biograph mMR MRI and a pQCT (Stratec Medizintechnik XCT2000). If the participant was unsure of which knee had more pain, the non-dominant leg was scanned. Anteroposterior knee X-rays were obtained in coronal and sagittal views for radiologist grading using Kellgren-Lawrence (KL) scores.

**pQCT acquisition:** Knee circumference (at points 10 cm superior and inferior to the base of the patella) was assessed (<40 cm) to ensure sufficient fit into the gantry. Participants were positioned in the pQCT scanner with the base of the patella centered to the laser with sufficient Z-distance in either direction for scans and scout. Each of 4 separately acquired transaxial CT slices (2.3 ± 0.5 mm thick, 200 µm in-plane dimensions, 38 kVp X-ray energy) were prescribed at 1% of tibial length distal to the tibial plateau and 2% of femoral length proximal from the femoral condyles for medial and lateral compartments (Fig. 1A & 1B). These scout positions were selected based upon motivation from scans of the distal tibia and radius at 4% with some empirical adjustments to arrive at a site just interior to the cortical shell of the subchondral region. Scan speed was set at 10 mm/*sec*. Effective dose was 1 µSv per slice.

**Fig. 1A:**
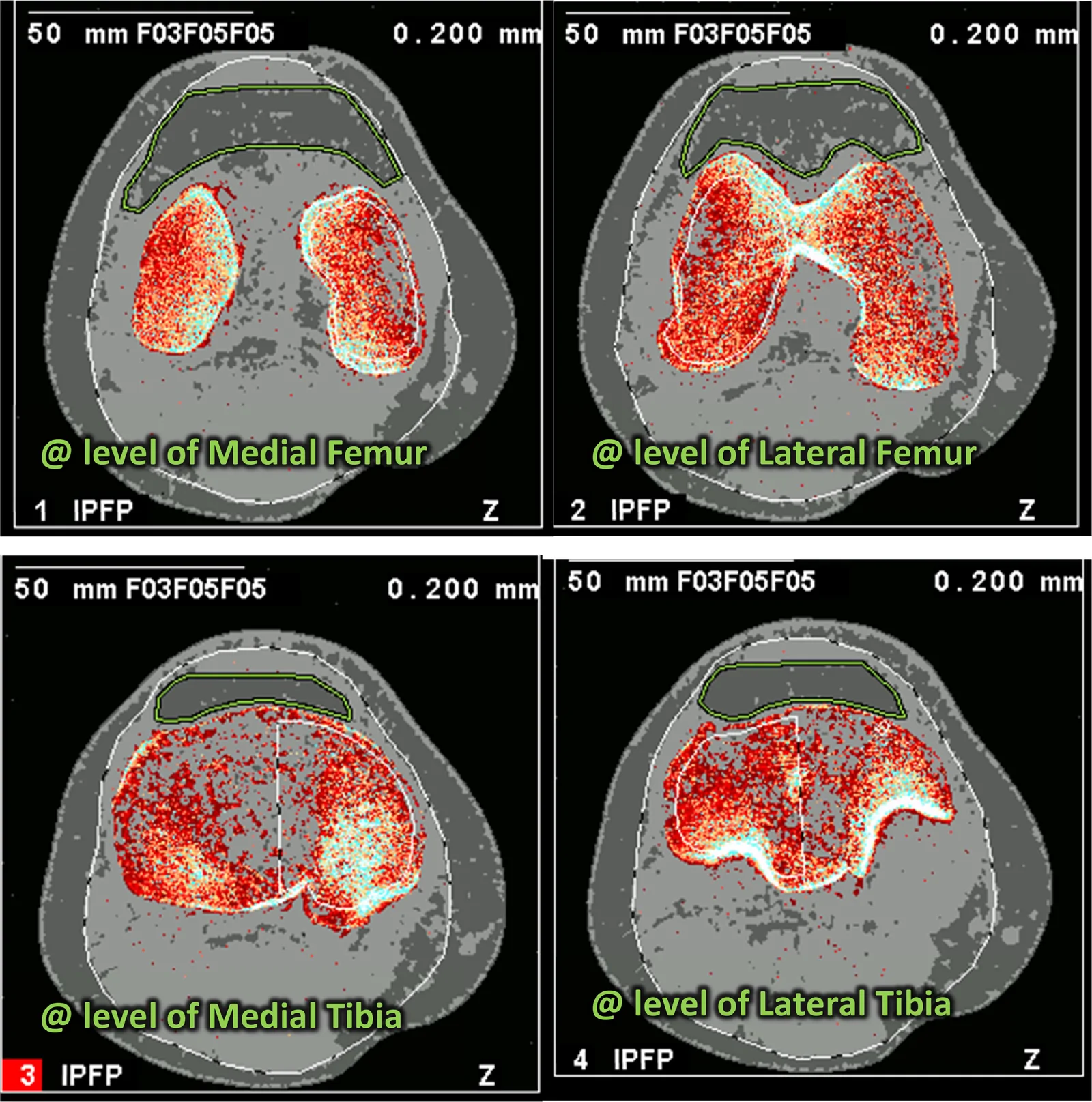
Sample from a pQCT transaxial slice demonstrating segmentation boundaries (green) for the IPFP. The image on the left shows a transaxial slice at the medial femoral level and the image on the right shows a slice at the lateral tibial level.

**Fig. 1B:**
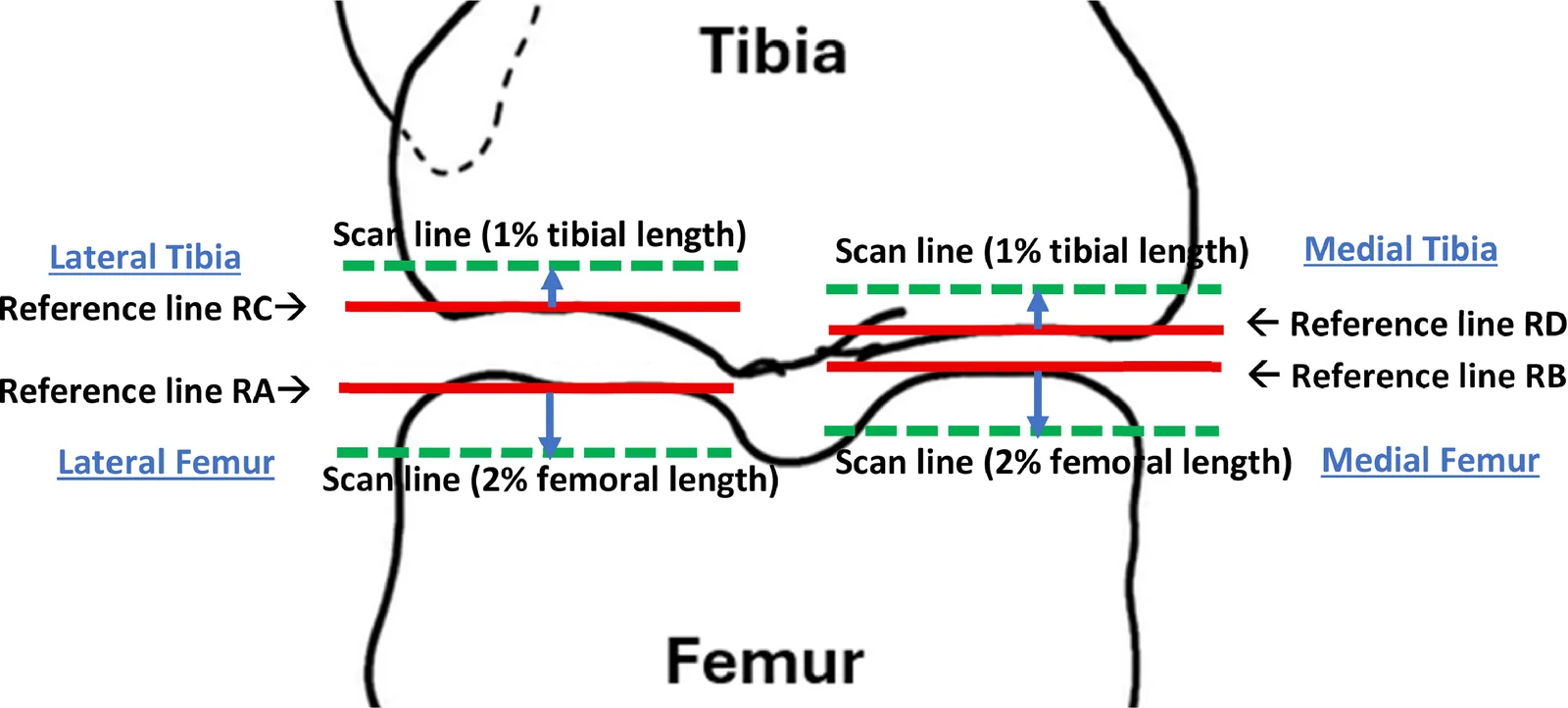
Scout markers for the 4 transaxial slices in pQCT imaging. Markers were aligned at both medial and lateral condyles of the femur and tibia at distances equal to 2% of femoral length for femoral condyle references (RA, RB for lateral and medial condyles, respectively); and equal to 1% of tibial length for tibial plateau references (RC, RD for lateral and medial condyles, respectively).

**pQCT Segmentations:** IPFP within each pQCT slice was manually segmented using XCT 5.20C Analysis Software (Fig. 1A). The IPFP appears as a darker grey (less dense) region between the patella and tibia/femur, and embedded among periarticular soft tissues (light grey). Rough contours around the IPFP were loosely defined within the periarticular soft tissues avoiding bone in each of the 4 separately acquired transaxial slices (medial femoral, lateral femoral, medial tibial, and lateral tibial), careful not to miss islands of fat presumed to be part of the IPFP. These labels refer to distinct acquisition locations rather than subdivisions within a single image. An inner threshold of −50 mg/cm^3^ and outer threshold of 40 mg/cm^3^ was applied to separate periarticular soft tissues from the IPFP. Cross-sectional area (CSA) and mass were extracted after applying a series of median filters (F03F05F05, a 3 × 3 median filter with −500 to 500 mg/cm^3^ threshold limit, and two 5 × 5 median filters with −500 to 300 mg/cm^3^ threshold limit). Outputs were obtained within the 'trabecular' mask section since cortical areas were not applicable in this soft tissue analysis. An example can be seen of tibial and femoral slices in Fig. 1A. IPFP density was also computed as mass of the segmented IPFP tissue over its volume, as defined by the product of its CSA and the slice thickness. This metric putatively represents the degree of IPFP fibrosis, a known downstream consequence of inflammation [18]. All IPFP parameters were examined per slice or summated over all slices.

**MRI acquisition:** A proton density-weighted (PDW) MRI in sagittal view (0.4 × 0.4 × 3.0 mm voxel size, TE/TR: 35/3140 ms, flip angle: 150°) was performed by a technologist using a quadrature knee coil, obtaining 25 slices centred about the knee joint. Ten contiguous slices were selected for analysis, encompassing the full volume of the IPFP in all planes.

**Fig. 2:**
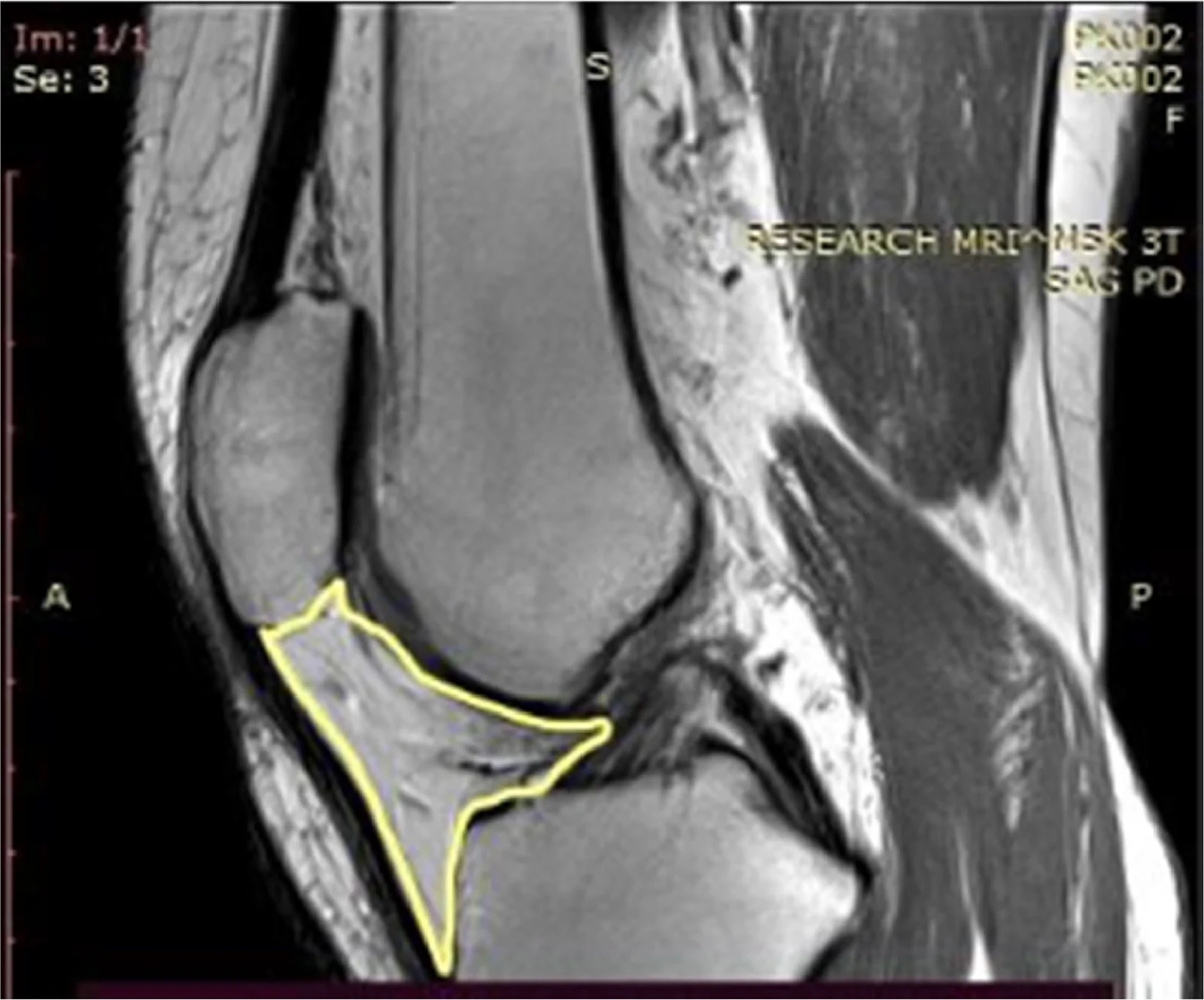
Sample image from a sagittal view PDW MRI demonstrating the IPFP boundaries (yellow).

**MRI Segmentations:** Manual segmentation of the MR images was performed using Slice-o-matic for each sagittal slice containing the IPFP on visual inspection (Fig. 2). Slice-wise volumes were computed into a final volume by summating data across all slices (direct calculation) and applying software interpolation. The volume from MRI was also calculated manually to see whether there was any difference in associations with the knee symptoms and if the interpolation formula from the software provides a uniform volume with our segmentations. The medial and lateral inferior genicular artery connects through the IPFP, so where visible, this structure was excluded.

**Statistical analyses:** Multivariable linear regression models examined the relationship between the IPFP parameters (exposures) with each KOOS subscale, ICOAP intermittent and constant pain, or physical function tests (outcomes), reporting unstandardized regression coefficients (B) and associated 95% confidence intervals. The pQCT slice-wise IPFP CSAs measured at the level of the four compartments (medial tibial, lateral tibial, medial femoral, lateral femoral) were each examined for associations with the above outcomes. These pQCT IPFP CSAs were also compiled into a total volume, factoring in slice thickness, for a separate analysis. This was done to see if the collective metrics had a greater impact on pain or whether there were particular anatomical regions that were more impactful. For MRI, only the total volume from PDW images was examined as an exposure. The primary *a priori* analysis was to study overall IPFP size and density’s association with knee pain (overall, intermittent, or constant). All other analyses were considered secondary. Participants’ age, BMI, use of pain medications, and KL score (≥2 vs <2) were included as covariates in each of the models. Collinearity of variables was evaluated using variance inflation factors. Holm’s correction was applied to account for multiplicity for all secondary analyses. A paired *t*-test was used to compare differences in IPFP areas (medial and lateral combined) between femoral (more superior) and tibial (more inferior) regions. Furthermore, a paired *t*-test was conducted to compare IPFP volumes determined by manual calculation versus interpolation by the Slice-o-matic software. All tests were performed at the 95% confidence level using SAS v9.4.

## Results

Participants (N = 47) had a mean age of 61.2 ± 8.8 yrs and a mean BMI of 22.47±3.20 kg/m^2^ in the non-obese range (Table 1). The KL score of knee X-rays showed 27.66% of participants had established OA (KL≥2) (Table 5). None of these participants had knee trauma as surveyed. The visible IPFP CSAs from pQCT imaging was on average larger when acquired at the level of the femoral compartments than at the level of the tibial compartments (mean difference of 75.8 mm^2^, p-value <0.001) (Table 3).

**Table 1:** IPFP area and volume characteristics from pQCT and MRI scans. A lower KOOS score or a higher ICOAP score indicate more painful responses. KOOS and ICOAP scores range from 0–100.The ICOAP scores are normalized to a 0–100 scale to match KOOS score ranges. A lower KOOS score or a greater ICOAP score indicate poorer patient outcomes. M= medial, L= lateral, F= femoral, T = tibial.

Variable	N	Mean	SD	Min	Max
**Age (years)**	51	61.16	8.82	50.00	83.00
**BMI (kg/m^2^)**	51	22.47	3.20	14.44	29.32
**ICOAP Intermittent %**	49	20	21	0	75
**ICOAP Constant %**	50	11	21	0	85
**KOOS Pain %**	50	81	16	42	100
**KOOS Activities of Daily Living (ADL) %**	50	90	13	41	100
**KOOS Quality of Life (QoL) %**	50	65	27	13	100
**pQCT-derived IPFP parameters**					
**Total IPFP Area (cm^2^)**	49	19.98	4.06	12.48	28.82
**MF IPFP Area (cm^2^)**	49	7.31	1.72	3.41	11.30
**LF IPFP Area (cm^2^)**	49	6.47	1.80	2.53	11.40
**MT IPFP Area (cm^2^)**	49	2.56	1.01	0.39	4.63
**LT IPFP Area (cm^2^)**	49	3.64	1.10	0.68	6.25
**MRI-derived IPFP parameters**					
**Volume calculated (cm^3^)**	43	13.91	3.05	7.65	22.86
**Volume interpolated (cm^3^)**	43	16.57	3.65	9.06	27.26

**Fig. 3:**
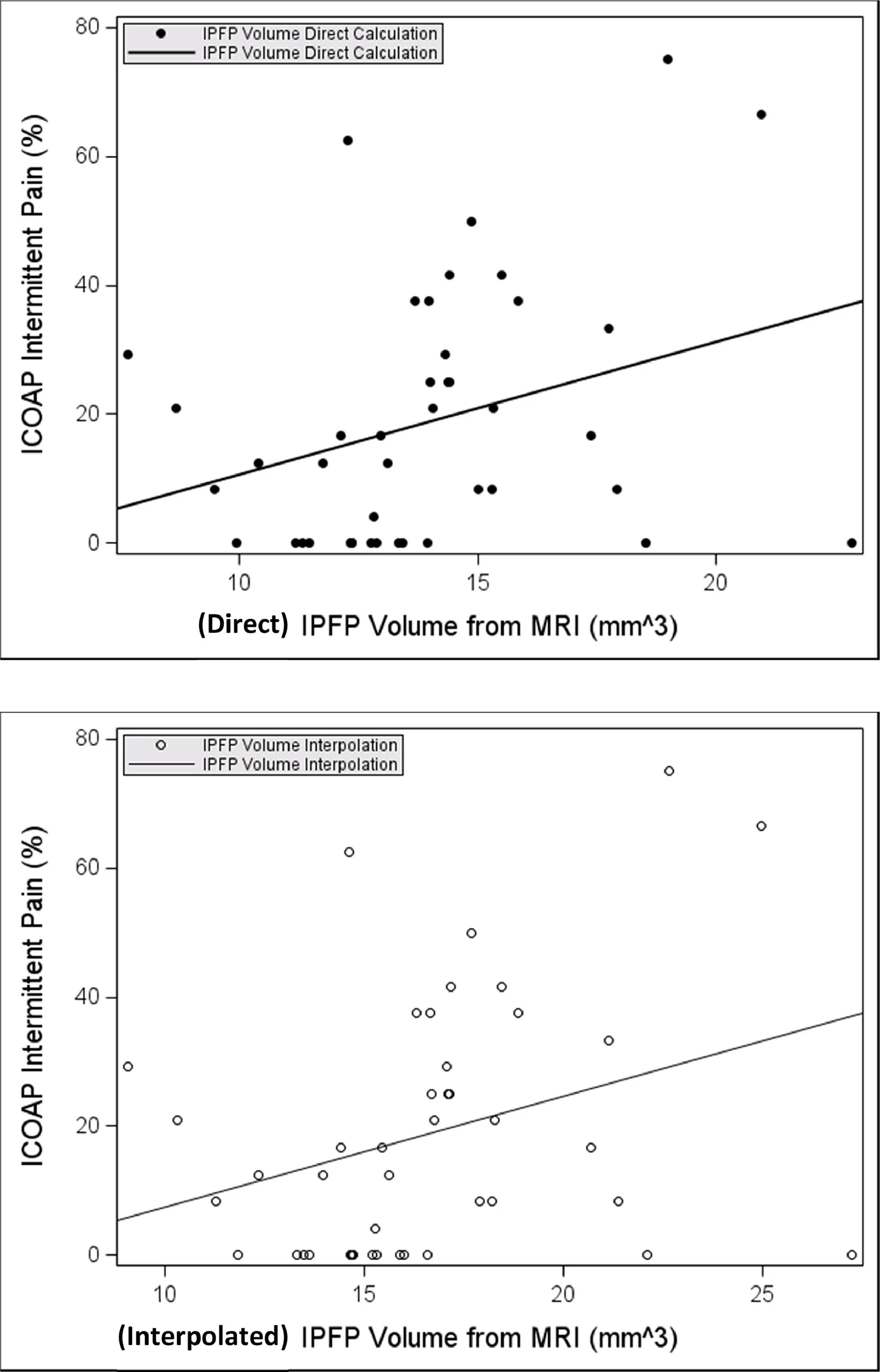
MRI-derived IPFP volume (residual after regressing on age, BMI, use of pain medications, and KL score≥2) relationship with ICOAP intermittent pain (both direct calculation and interpolated values).

For our primary analysis, calculated IPFP total volume from MRI was associated with nearly a 2% higher ICOAP intermittent score while the interpolated volume from MRI was associated with a 1.67% higher ICOAP intermittent score (Fig. 3). A similar effect was observed for KOOS pain, KOOS ADLs, and ICOAP total pain, but with smaller effect sizes (around 1%) that only reached marginal significance levels (p < 0.10) (Supplementary Files 1). These results indicate that a larger overall IPFP volume derived from MR images translate to worse knee pain outcomes, consistent with our original hypothesis. However, they contrast with most region-specific analyses derived from pQCT (Table 2).

**Table 2:** General linear regression model associations of significance between IPFP areas/volumes and knee pain scores. Bolded rows were significant at the p < 0.05 level while underlined rows were marginally significant with p-values <0.10. M= medial, L= lateral, F= femoral, T = tibial.

Exposures	Outcomes	N	Estimate	Standard Error	P-value	95% Confidence Interval	
***pQCT-derived associations***							
**MF IPFP Area (cm^2^)**	**KOOS Pain**	47	0.025	0.013	0.074	−0.003	0.052
**MF IPFP Area (cm^2^)**	**KOOS ADL**	47	**0.023**	**0.011**	**0.038**	**0.001**	**0.044**
**MF IPFP Area (cm^2^)**	**ICOAP Constant Pain**	47	**−0.047**	**0.017**	**0.010**	**−0.083**	**−0.012**
**MF IPFP Area (cm^2^)**	**ICOAP Intermittent Pain**	47	−0.031	0.017	0.071	−0.065	0.003
**MF IPFP Area (cm^2^)**	**ICOAP Total Pain**	47	**−0.039**	**0.016**	**0.017**	**−0.070**	**−0.007**
**LF IPFP Area (cm^2^)**	**9-step Stair Climb (s)**	47	**−0.007**	**0.003**	**0.032**	**−0.014**	**−0.001**
**MF IPFP Area (cm^2^)**	**9-step Stair Climb (s)**	47	**−0.007**	**0.003**	**0.050**	**−0.014**	**0.000**
**MT IPFP Area (cm^2^)**	**40m walk (s)**	47	−0.794	0.455	0.088	−1.713	0.124
**LT IPFP Density (mg/cm^3^)**	**KOOS ADL**	47	**−4.267**	**1.697**	**0.016**	**−7.694**	**−0.841**
**LT IPFP Density (mg/cm^3^)**	**KOOS QOL**	47	−6.258	3.331	0.068	−12.986	0.470
***MRI-derived associations***							
**Calculated MR IPFP Volume (cm^3^)**	**KOOS Pain**	43	−1.278	0.686	0.070	−2.667	0.111
**Calculated MR IPFP Volume (cm^3^)**	**KOOS ADL**	43	−1.148	0.586	0.058	−2.334	0.039
**Interpolated MR IPFP Volume (cm^3^)**	**KOOS Pain**	43	−1.069	0.574	0.071	−2.231	0.094
**Interpolated MR IPFP Volume (cm^3^)**	**KOOS ADL**	43	−0.959	0.490	0.058	−1.952	0.034
**Calculated MR IPFP Volume (cm^3^)**	**ICOAP Intermittent Pain**	43	**1.996**	**0.834**	**0.022**	**0.307**	**3.686**
**Calculated MR IPFP Volume (cm^3^)**	**ICOAP Total Pain**	43	1.492	0.805	0.072	−0.140	3.123
**Interpolated MR IPFP Volume (cm^3^)**	**ICOAP Intermittent Pain**	43	**1.669**	**0.698**	**0.022**	**0.255**	**3.083**
**Interpolated MR IPFP Volume (cm^3^)**	**ICOAP Total Pain**	43	1.248	0.674	0.072	−0.117	2.613

**Table 3:** Paired T-tests: 1) Comparing the IPFP areas from slices at the level of the femoral condyles and tibial condyles. The femoral and tibial areas were calculated by the sum of medial and lateral slices. 2) Comparing volumes retrieved from interpolation by the segmentation software versus manual calculation. Bolded rows were significant at the p-value <0.05 level.

Tested Paired Comparison:		N	Mean Difference	95% CL		Std Dev	P-value
**IPFP Area Femoral slices pQCT (mm^2^)**	**IPFP Area Tibial slices pQCT (mm^2^)**	49	**75.84**	**65.66**	**86.01**	**35.42**	**<0.001**
**Interpolated MR IPFP Volume (cm^3^)**	**Calculated MR IPFP Volume (cm^3^)**	43	**−2.66**	**−2.84**	**−2.48**	**0.60**	**<0.001**

**Fig. 4:**
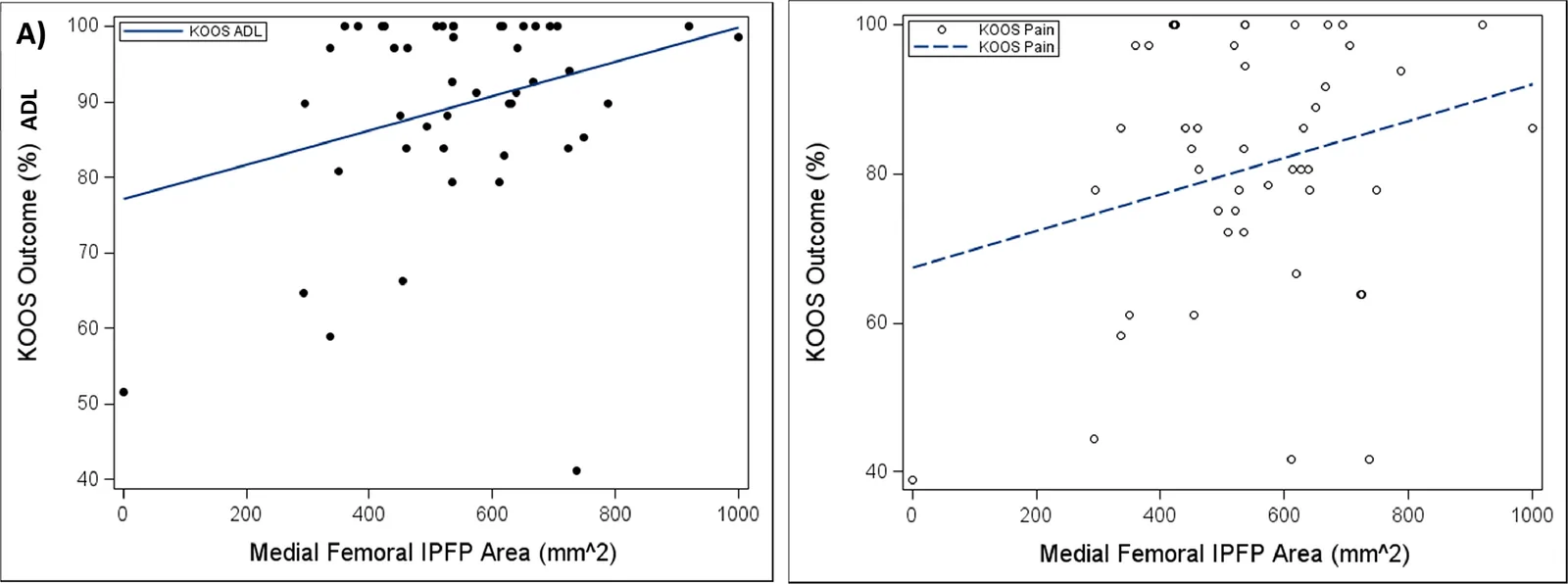

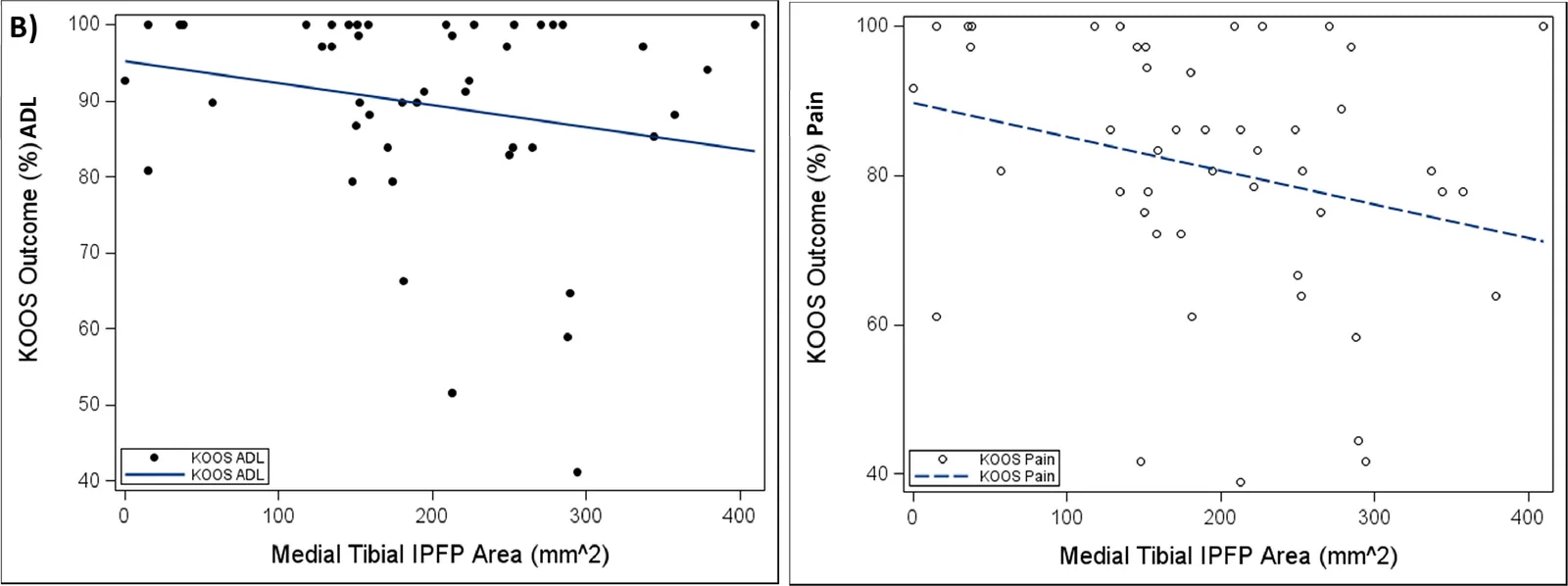
pQCT-derived IPFP CSA measured at the level of the A) medial femur and B) medial tibia and its relationship with KOOS subscale scores (ADLs (top panel) and Pain (bottom panel)).

**Fig. 5:**
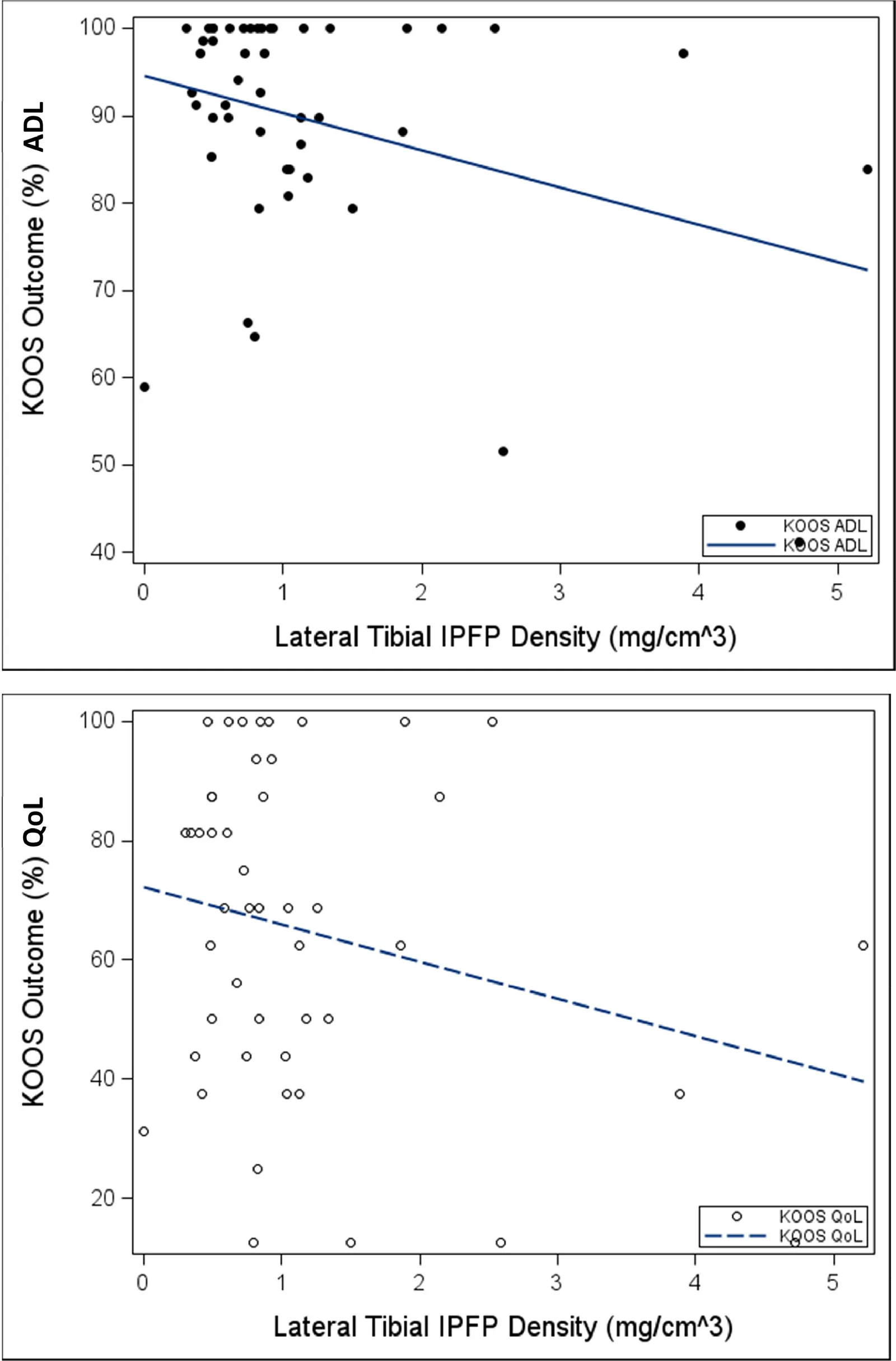
pQCT-derived IPFP density measured at the level of the lateral tibia and its relationship with KOOS subscale scores (ADLs (top panel) and QOL (bottom panel)).

In regional pQCT analyses, a 1 cm^2^ larger IPFP CSA measured at the medial femoral compartment level was associated with a 2.3% higher KOOS ADL score, a 4.7% lower ICOAP constant score, and a 3.9% lower ICOAP total score (Table 2). These results indicate that a larger regional IPFP CSA may be linked to having less severe knee symptoms. Although only marginally significant, a larger IPFP area at the medial femur also related to less severe KOOS pain (B = 2.50(−0.50,3.60)%) and ICOAP intermittent pain (B = −3.1(−6.5,0.3)%) (Supplementary Files 1). In contrast, at the medial tibia, there was an apparent relationship between larger IPFP area and more severe KOOS Pain (B = −4.6(−9.3,0.20)% per 1cm [2]) that was consistent with our original hypothesis (Fig. 4), but was not statistically significant. In terms of IPFP density, potentially representing the presence of fibrotic inflammation, a 1 mg/cm^3^ denser IPFP tissue, as measured at the level of the lateral tibial compartment, was associated with a 4.3% lower KOOS ADL score suggesting significantly worse symptoms (Fig. 5). A similar relationship was observed for KOOS QOL but this was only marginally significant (6.3% lower) (Table 2). Since these were secondary analyses, Holm’s correction was applied but none of the originally significant associations remained robust enough against multiple testing probability thresholds.

While the total fat pad volume for MR images was correlated with worse intermittent knee pain, the total volumes formulated from summation of individual pQCT slices did not reveal a correlation with outcomes. Other secondary analysis models did not produce statistically significant results (Supplement Files 1).

When comparing the two different modalities for imaging the knee, MRI-derived volumes and pQCT-derived areas were moderately to strongly correlated (Table 4). A comparison of the manually calculated versus interpolated volume calculations from the MR images indicate that although there was a significant difference (Table 3), they were systematically linear, meaning the manufacturer software applied a constant in its formula for volume calculation. This was corroborated by the consistent associations found for both metrics in terms of their relationship with knee symptom outcomes.

**Table 4:** Correlation of IPFP volumes and areas retrieved from MRI versus pQCT as measured by Pearson correlation coefficients (r).

MRI Volume Measure	pQCT IPFP Measure	N	Pearson r	p-value
**Interpolated MR IPFP Volume**	**MF IPFP Area**	42	0.400	**0.009**
**Interpolated MR IPFP Volume**	**LF IPFP Area**	42	0.548	**<0.001**
**Interpolated MR IPFP Volume**	**MT IPFP Area**	42	0.275	**0.078**
**Interpolated MR IPFP Volume**	**LT IPFP Area**	42	0.426	**0.005**
**Interpolated MR IPFP Volume**	**IPFP Total Area**	42	0.587	**<0.001**
**Calculated MR IPFP Volume**	**MF IPFP Area**	42	0.401	**0.009**
**Calculated MR IPFP Volume**	**LF IPFP Area**	42	0.548	**<0.001**
**Calculated MR IPFP Volume**	**MT IPFP Area**	42	0.276	**0.077**
**Calculated MR IPFP Volume**	**LT IPFP Area**	42	0.427	**0.005**
**Calculated MR IPFP Volume**	**IPFP Total Area**	42	0.587	**<0.001**

**Table 5:** KL grade frequencies for all participants.

KL Score	Frequency	Percent (%)	Cumulative Frequency	Cumulative Percent (%)
**0**	9	19.15	99	19.15
**1**	25	53.19	34	72.34
**2**	10	21.28	44	93.62
**3**	3	6.38	47	100.00

## Discussion

The significance of this research lies in this previously under-studied cohort of postmenopausal women with knee pain who are non-obese, thus limiting the potential influence of systemic sources of fat and load-bearing on knee pain [13,17,21,22]. These results show that despite accounting these factors, a larger overall volume of IPFP as measured by MRI was associated with greater intermittent knee pain, while thinner IPFP cross-sections at the level of femoral compartments may reflect worse knee pain scores. Remarkably, a higher IPFP density, suggesting the presence of fibrosis and its underlying inflammation, was also linked to having worse knee symptom outcomes. While total IPFP volume estimated by pQCT correlated well against MRI, it lacked overall clinical significance unless examined by region.

One key finding of this study is the association between greater IPFP volume and having worse intermittent knee pain. This observation confirms results from *Bohnshack* et al. who reported that patients with anterior knee pain experienced an intermittent, burning peripatellar pain that may be associated with larger IPFPs [12]. While our study did not measure anterior knee pain, we were able to relate IPFP volume to a variety of general knee symptoms including intermittent knee pain, ADLs, and overall activity-related knee pain, which were exploratory and were unable to localize to a specific IPFP pain origin. Our results also corroborate reports by *Cowan* et al. who found larger IPFP volumes in OA patients than controls. However, they did not link these differences to variable experiences in pain [23]. Other reports also found that OA patients generally indicated either a dull, aching pain that is constant or an intermittent pain that varies in intensity, commonly associated with anterior knee pain syndrome [10,24]. A substantial portion of sports-related injuries involving the knee are also linked to anterior knee pain [11,12]. Despite the older age of our cohort, some were still active, so this possibility may be relevant. While adjustment for KL grade helped account for radiographic OA severity, it did not establish independence from all OA-related structural contributors to pain, particularly in participants with more established disease whose pain could originate from different sources and could affect the interpretation of our study findings.

Morphologically, the IPFP appears to vary across individuals, as shown by large ranges in both CSA and volumes (Table 1) in our study. The larger IPFP size could have greater secretory potential, allowing more inflammatory cytokines and adipokines into the synovium, but this may perhaps be triggered only during an inflammatory state. Leptin levels are already increased in women with knee OA, but interestingly, not in men [22], which could indicate different mechanisms for inflammation-driven pain experiences in women versus men. While we did not examine men in our study for comparison, results of our study motivate future sex-stratified analyses linking IPFP size and inflammatory players with knee OA outcomes. Interpretation of IPFP size and its associations with knee symptoms should consider the potential confounding effects of systemic obesity and body size. Prior literature suggests that IPFP dimensions may reflect both body size and adiposity-related factors [25], although these relationships are inconsistent across cohorts. We therefore adjusted for BMI as a covariate representing systemic adiposity and body size, rather than applying both knee ratio-based normalization and body size adjustments, while acknowledging that BMI may not fully capture knee size.

The combined morphometric results here suggest that IPFP volume is a correlate of knee pain that may depend not just on its size but its shape configuration. The fact that larger volumes yet thinner cross-sections were concurrently related to different measures of knee symptoms suggest that (supero-inferiorly) long and skinny IPFP phenotypes maybe unfavourable to knee OA outcomes. Conversely, it is possible that short yet wide IPFP shapes may be associated with less knee symptoms potentially by virtue of better mechanical support. However, larger studies are necessary to validate these combinations as a risk factor for knee OA. As the IPFP is already visible within standard of care PDW knee MRIs that are occasionally ordered pre-arthroscopically, future studies could opportunistically integrate this information to retrospectively assess its prognostic value, particularly for those whose weight may not be a major contributor to knee damage.

With the increasing investigation of knee OA using CT [26], future research studies employing CT could also capitalize on the ability to evaluate IPFP cross-sectional areas and density, given its consistent correlation with KOOS outcomes shown here. We observed some associations when IPFP cross-sectional area was measured at the level of the femoral plateau, which may be explained by the fact that the IPFP appears to be largest or most variable at this location; however, it had weaker effect sizes compared to slices at the tibial plateau. This smaller effect could be due to the fact that although the area measured at the femoral condyles is larger than at the tibia (as shown in Fig. 1A), the boundaries of the latter are better defined. Therefore, the sparse borders of the femoral-level IPFP may introduce uncertainty and obscure the ability to observe consistent and larger correlations with knee symptoms.

The relationship between a denser IPFP and having worse knee symptoms shown in our study was a unique discovery. It may be explained by the presence of fibrosis, which is known to be triggered by underlying inflammation [18]. Given these established correlative links and prior mechanistic support, we believe IPFP density may have potential to serve as a surrogate marker for inflammation using pQCT. An et al. demonstrated that reversal of fibrotic changes in the IPFP alleviates pain and reverses articular cartilage degeneration in mice [27]. This association was also confirmed in a study of 874 older adults with the presence of IPFP hypointensity within T2-weighted fat-saturated fast spin echo MRI, representing fibrosis being linked to more cartilage defects, osteophytosis, and overall knee symptoms [28]. Being able to represent these fibrotic changes using IPFP density would mean that we could easily track similar intervention-related effects with minimal ionizing radiation on a small modality footprint. While no previous studies have been performed using pQCT or high resolution pQCT to quantify fibrosis within the IPFP, studies in the cancer literature support using CT to measure fat attenuation as a surrogate for fibrotic changes, which have been linked to poorer survival and tumour recurrence [29]. In view of the symptomatic link of multiple tissue properties to IPFP in the context of OA, there is clear value in applying multi-modal investigation to study the IPFP. This approach would allow the multi-factorial role of IPFP on knee OA outcomes to be captured more completely, with IPFP density serving as a potential surrogate marker for inflammatory insult. In fact, a previous review summarized the biphasic nature of the IPFP on knee OA outcomes [30], which has been shown to vary with anatomical location, as supported by the region-specific variations described in our study.

Given the cross-sectional nature of this study, caution should be applied to the interpretation of results, as findings do not suggest any causal relationships. Other limitations in this study include not capturing gender and pain catastrophizing that could affect painful experiences. The convenience sampling used in this study could also bias towards a more health-seeking cohort. The sample size is smaller, limiting our ability to account for other potential confounders including physical activity as well as limiting the power of our analyses to observe significant effects. While the MRI-derived IPFP segmentation is crude and its reproducibility not established, it was not used for measuring change. However, stronger associations with other outcomes may be possible with a more reliable method of capturing IPFP morphometry automatically, including other perfusion related properties that could reflect its inflammatory potential. Indeed, automated MR segmentation of periarticular fat has now been well-refined [4,31] and algorithmic modifications to segment the IPFP could be a future reality, especially with the advent of neural networks and transformers. Another important limitation is that the present study emphasized IPFP morphometric measures and pQCT-derived density, whereas other potentially informative MRI features such as Hoffa-synovitis signal abnormalities, edema-like signal changes, and radiomic or texture-based descriptors were not assessed. Although semi-quantitative Hoffa-synovitis scoring has been reported previously in OA imaging studies, the MRI protocol used here was not optimized for standardized quantitative signal intensity comparisons across participants. Future studies using standardized fat-suppressed or quantitative MRI acquisitions may better characterize inflammatory and compositional features of the IPFP beyond morphometry alone. Accordingly, the present findings should not be interpreted as a comprehensive characterization of the IPFP’s symptomatic role, especially given the lack of measurement of anterior knee pain. These findings should also be interpreted cautiously. The MRI- and pQCT-derived parameters may capture different aspects of IPFP morphology and tissue composition, and the observed cross-sectional relationships do not establish mechanism. Given that the secondary associations did not remain statistically robust after correction for multiple testing, these results are best viewed as hypothesis-generating.

## Conclusions

In this cohort of non-obese post-menopausal women, the exploratory cross-sectional analyses identified a larger overall IPFP volume but smaller axial cross-sectional area as being related to greater knee symptoms including intermittent pain, suggesting a long and thin IPFP may be unfavourable to knee OA symptoms. Higher IPFP density may reflect fibrosis which may have an underlying origin from inflammation, and its consistent association with worse knee symptoms suggests that multi-modal imaging including MRI and CT may be warranted for better characterization of the IPFP. These findings are hypothesis-generating and should be interpreted cautiously. While the present study does not suggest any causal relations, it provides evidence to support larger future studies to disentangle IPFP’s association with knee symptom development.

## CRediT authorship contribution statement

**Nima Yazdankhah:** Conceptualization, Formal analysis, Investigation, Data curation, Writing – original draft, Writing – review & editing, Supervision, Project administration. **Vahid Anwari:** Funding acquisition, Formal analysis, Data curation, Writing – review & editing. **Siwen Liu:** Funding acquisition, Formal analysis, Writing – review & editing. **Emily Ha:** Funding acquisition, Formal analysis, Writing – review & editing. **Andy Kin On Wong:** Conceptualization, Methodology, Formal analysis, Resources, Data curation, Writing – review & editing, Visualization, Supervision, Funding acquisition.

## Declaration of competing interest

The authors declare that they have no known competing financial interests or personal relationships that could have appeared to influence the work reported in this paper.

## Appendix Supplementary materials

Image, application 1
